# Design of a Sensor-Based Digital Product Passport for Low-Tech Manufacturing: Traceability and Environmental Monitoring in Bio-Block Production

**DOI:** 10.3390/s25185653

**Published:** 2025-09-10

**Authors:** Alessandro Pracucci, Matteo Giovanardi

**Affiliations:** Levery S.r.l. Società Benefit, Via Pisino 66, 47814 Bellaria-Igea Marina, Italy; m.giovanardi@levery.it

**Keywords:** Digital Product Passport (DPP), bio-based construction materials, Internet of Things (IoT), traceability, Life Cycle Assessment (LCA), environmental monitoring, circular economy (CE)

## Abstract

The Digital Product Passport (DPP) is an emergent strategic tool poised to significantly enhance traceability, circularity, and sustainability within industrial supply chains, aligning with evolving European Union regulatory frameworks. This paper introduces a conceptual sensor-based DPP architecture specifically designed for the construction industry, exemplified by a real case study for a bio-based manufacturing company. This framework facilitates a transparent and accessible data management approach, crucial for fostering circular practices and guiding stakeholders in decision-making along the value chain. The proposed architecture addresses critical challenges in product-related traceability and information accessibility across the entire product life cycle, spanning from raw material supply to the construction and installation process (A1–A5 stages). Data collected from the low-tech sensor network and digital tools can generate relevant environmental indicators for Life Cycle Assessment (LCA) and DPP creation, thereby offering a comprehensive, detailed, and certified overview of product attributes and their environmental impacts. The study clarifies the benefits and current barriers to implementing a sensor-based DPP architecture in low-tech construction manufacturing, underscoring the potential of lightweight, interoperable sensing solutions to advance compliance, transparency, and digitalization in traditionally under-digitized sectors like construction materials manufacturing.

## 1. Introduction

The construction industry is under increasing pressure to adopt sustainable practices and improve supply chain transparency. This is driven by the evolving regulatory frameworks of the European Union [1,2], including the Ecodesign for Sustainable Products Regulation (ESPR) [3] and the Corporate Sustainability Reporting Directive (CSRD) [4]. In this context, the Digital Product Passport (DPP) is emerging as a strategic instrument to enable traceability, circularity and environmental accountability across industrial supply chains and will become compulsory in the upcoming years [3,5]. However, implementing DPP systems in traditional manufacturing sectors characterized by low-technology production environments presents significant technical and economic challenges, limiting widespread adoption [6,7,8]. An analysis of the existing literature on DPP for construction products reveals a significant gap, as the topic has not yet been fully integrated and harmonized by European and national standards. Although various working groups and academics are developing guidelines and standardized processes for data management and sharing, there is currently no existing research that could support a comprehensive literature review of the current context. These challenges are particularly evident in the construction materials manufacturing sector, where production processes often rely on manual data collection methods and low-digitalized processes [9,10,11]. This results in operational inefficiencies and compromised data accuracy in sustainability reporting [12,13]. This is particularly evident in the bio-based construction materials industry, where manufacturers have successfully commercialized products such as hemp-based building blocks while maintaining traditional, low-digitization production workflows [14]. Furthermore, using wasted materials from different supply chains and cross-sectorial industries necessitates greater attention to the quality of the resources used [15]. Current practices involve spreadsheet-based data collection systems and manual verification protocols, which create barriers to the systematic integration of life cycle data required for comprehensive DPP implementation. Recent advances in Internet of Things (IoT) technologies, Industrial Internet of Things (IIoT), and low-cost sensing solutions present opportunities to close the digitalization gap [16,17,18]. However, the existing literature reveals significant differences in the approaches to sensor integration for manufacturing traceability. While some studies advocate comprehensive, high-specification sensor networks with advanced analytics capabilities [19,20], others emphasize the importance of lightweight, interoperable solutions that can be retrofitted into existing production environments without substantial infrastructure modifications [21,22,23]. Furthermore, several papers on the adoption of IoT and IIoT in production and manufacturing sectors report that these devices often have limitations, such as narrow bandwidth, signal interference, and a limited transmission range. These inherent drawbacks make them vulnerable to various external factors. For example, they can be negatively affected by the environmental conditions of production sites, interference from other devices, or the factory structures themselves. These limitations are crucial to consider when designing and implementing a reliable and secure data collection system [24,25]. This controversy is particularly relevant for small and medium-sized enterprises (SMEs) in the construction sector, where investment constraints and limited technical expertise necessitate carefully calibrated technology adoption strategies [24]. From an economic perspective, the hardware of IoT and IIoT is often low-cost, and many smart systems are already in use, meaning the primary investment lies in the systematic collection and management of the data. The cost incidence strongly depends on the overall system architecture, the number of connected devices, the quality of the sensors, and the frequency with which data is collected [26]. This study addresses the critical gap between the best practices in tracking and collecting product-related life cycle data promoted by DPP regulations and the practical constraints of implementing them in low-technology manufacturing environments. The research presents a conceptual framework for a sensor-based DPP systems designed for bio-based construction products.

This work’s main contribution is demonstrating how lightweight, interoperable IoT sensing solutions can enable real-time DPP data collection in traditionally under-digitized manufacturing environments, providing a comprehensive overview of products’ supply chains and impacts. This supports compliance with emerging EU regulatory frameworks and promotes local circular economy models through agricultural waste valorization. The findings provide a scalable framework that enables SMEs in the construction materials sector to adopt digital traceability systems without compromising operational efficiency or requiring substantial capital investment in advanced manufacturing infrastructure.

## 2. Materials and Methods

This research employed a case study methodology to investigate the implementation of a sensor-based DPP within a low-tech manufacturing environment in the construction sector. This approach was selected to provide an in-depth, holistic understanding of the complex interplay between technological integration, existing operational practices, and specific industry challenges.

### 2.1. Case Study Description

The case study focused on a bio-block manufacturing company in Southern Italy. The company is representative of the small and medium-sized enterprises (SMEs) prevalent in the European bio-based manufacturing construction sector. The firm specializes in producing hemp-based bio-blocks and is working toward integrating local agricultural waste aggregates to improve the product’s mechanical and environmental performance. A key challenge for the company is its supply chain. Due to limitations and frequent changes in cultivation regulations in Italy, the hemp must be imported from abroad, which significantly impacts the bio-block’s overall environmental footprint. For this reason, the company is investigating the possibility of integrating local bio-based aggregates, such as olive stones and almond shells, as there is strong local production in the area of the business. This provides a significant local supply of agricultural waste, which could be used as a primary aggregate in the production cycle.

**Figure 1:**
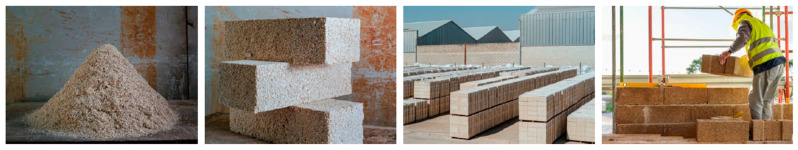
Bio-block production process images (left to right: raw material, bio-block, warehouse, and construction site) (credit: ©BIOmat Canapa).

The bio-blocks are produced through simple, low-level industrialized processes: mixing the main components, molding them in casts, and natural air-drying (Figure 1). The drying process takes place in a factory that has been repurposed from an old brick manufacturing plant. Thanks to the area’s favorable environmental conditions, the facility does not require air conditioning or heating for the production plant. Once the bio-based products are made, the company markets its products throughout the entire country.

### 2.2. Methodological Framework

Given the lack of specific literature on DPP design within the construction sector, particularly concerning low-digitization manufacturing environments, a traditional literature review was not feasible. Therefore, this study employed a hybrid methodological framework to address this gap. The methodological framework comprised several key steps:

- Step 1. User story identification and process analysis through user-centered design. Initial investigations involved unstructured interviews to gather user stories from the manufacturer. These focused on current data collection practices, information sharing challenges, and operational pain points, deliberately avoiding bias towards specific technologies. This qualitative data informed a systematic documentation of the bio-block manufacturing workflow, from raw material inbound to outbound logistics, identifying critical data collection points and bottlenecks. Concurrently, an assessment of existing IT infrastructure (e.g., spreadsheets, ERP systems) was performed to establish baseline digitization levels and identify integration constraints. User stories were subsequently translated into functional and technical requirements.
- Step 2. Data-driven requirements analysis and circular data flow framework. A comprehensive data flow analysis was conducted to identify quantitative metrics and data sources, including manual records and fragmented digital information. This involved analyzing existing data collection workflows for inefficiencies and evaluating data quality, accessibility, and standardization for DPP implementation, with a focus on real-time monitoring opportunities. The methodology incorporated a life cycle-oriented approach (A1–A5) based on the EN 15978 standard, analyzing existing product certification schemes (such as EPDs) to identify data standardization patterns. The EN 15978 standard outlines the methodology for assessing the environmental performance of buildings, including life cycle stages from raw material extraction to end-of-life disposal. This informed an ontological architecture defining user roles, data sources, and information flows, following the European Commission’s product classification for construction [5].
- Step 3. DPP architecture design. The DPP’s technical architecture was designed using a hybrid approach, combining layered, microservices, and event-driven patterns. This addressed low-technology manufacturing constraints while ensuring scalability and maintainability. A five-layer structure (user interface, business intelligence, data processing, integration, data storage) provided modularity. Independent microservices for core functionalities (e.g., supplier data, Global Warming Potential calculations) allowed for selective adoption. Event-driven capabilities supported continuous monitoring and responsiveness for real-time data collection.
- Step 4. Hardware and software integration assessment. A robust methodology was developed to assess the integration requirements between the proposed DPP and the existing manufacturing IT infrastructure. This involved evaluating compatibility across data management systems, user interfaces, and operational workflows, and identifying business opportunities for IT system integration. Technical criteria included interoperability, data security, user accessibility, and scalability for SMEs. The assessment also identified minimum viable, affordable, and desirable sensing solutions that could integrate into existing production workflows with minimal infrastructure modifications, emphasizing compatibility with current spreadsheet-based systems and ERP integration to minimize adoption barriers. At the time of the investigation, eight types of sensors were evaluated. Although it is not possible to determine a priori the number and type of sensors to be used for a case study, these are the most common for monitoring and describing the manufacturing process of a construction product. The optimal number of sensors depends on factors such as production complexity, data granularity requirements, and budget constraints. Further investigations will be needed to determine the minimum number of sensors required per type of product.

## 3. Results

The design of the sensor-based DPP architecture resulted from four main analyses: the identification of specific information requirements for key actors in the product value chain, a detailed analysis of current data management practices in the manufacturing process, current product life cycle data availability, and the potential integration of sensors for automated data collection. Although the research was conducted on a specific case study with its own unique requirements, processes, and operational context, the findings and the approach can be considered significant and scalable to a large number of low-tech European manufacturing SMEs involved in the construction sector.

### 3.1. User Stories and Technical Requirements

The user-centered design methodology involved interviews of four company personnel (director, CFO, production engineer, project manager) who were directly involved in the managing and implementation of the bio-block. The interviews that were conducted defined 31 distinct user stories across the stakeholder categories, reflecting the complex requirements landscape for DPP implementation in bio-based construction materials manufacturing; the main technical requirements identified are reported in Appendix A. The finalized analysis demonstrates that strategic sensor deployment can address nines critical user stories with varying complexity levels and significant business impact potential. Table 1 presents the user stories with identified sensors and integration with DPP features, with an early analysis related to the complexity of implementation balancing tech adoption with business opportunities.

### 3.2. Process Workflow and Data Collection Framework

Based on the bio-block manufacturing company analysis and interviews, the bio-block production process workflow was systematically mapped to identify critical data collection points and sensor integration opportunities. Similar to the previous analysis (as detailed in Section 3.1), the information extracted from process analysis and interviews was organized according to the potential implementation of sensors and characterized by the complexity level. Table 2 presents the structured phases of the manufacturing process with relevant technical features of data flow management and automation opportunities. The company analyzed for the case study uses simplified Product Life Cycle Management (PLM) and Enterprise Resource Planning (ERP) systems for product design and business process organization. The internal PLM software is, therefore, mainly used for managing technical documents and integrating with work tools like CAD instruments. Meanwhile, the ERP systems in use govern the flow of materials (in–out) to produce bio-blocks and production management, facilitating operations and accounting. At the time of the study, the company had not adopted Supervisory Control and Data Acquisition (SCADA) or IIoT systems within its production plant. Outside the company, the exchange of information related to the bio-block product, such as product certifications and technical data, with suppliers and end customers (designers, builders, end users, etc.) mainly occurs through sharing documents via email or delivering paper-based documentation (e.g., transport documents).

### 3.3. Available Dataset and Data Integration Analysis

The current data landscape within the bio-block company manufacturing environment reveals a fragmented information ecosystem with significant opportunities for sensor-based DPP integration. Table 3 presents the comprehensive data inventory, identifying existing data sources, collection methodologies, and integration potential for the sensor-integrated DPP system for the different workflow phases. The data inventory highlights that while static and semi-static data, such as supplier information, material characteristics, and environmental impact data, are collected, they are largely managed through manual, fragmented methods like spreadsheets and physical documents. Furthermore, a critical gap exists in the collection of dynamic production process data, as key parameters like temperature, humidity, and energy consumption are not monitored. This lack of real-time data collection prevents effective process optimization, quality control, and accurate reporting of environmental impact, underscoring the need for a sensor-based system to bridge this information gap. It should be noted that these findings are specific to the bio-block case study and may vary for other bio-based products with different characteristics and processing methods.

### 3.4. IoT Sensor Integration and Automation Potential

The analysis of current manual data collection processes reveals significant opportunities for automation through minimum viable IoT and IIoT sensor deployment. Table 4 identifies the transition potential from manual to automated data collection, aligned with the proposed sensor-based DPP architecture. Key aspects of manual data collection are the potential for human error, data manipulation, and lack of transparency. The transition to automated, sensor-based systems significantly enhances the reliability and integrity of data. By capturing information directly from the source in real time, the risk of transcription errors and intentional data falsification is minimized. This is a critical factor for ensuring the accuracy and trustworthiness of the information that will be stored in the DPP, which must meet strict regulatory standards. Furthermore, implementing these systems requires a robust approach to cybersecurity. IoT and IIoT devices can be vulnerable to external threats, necessitating secure data transmission protocols and protected storage solutions. To ensure the confidentiality and integrity of the data throughout its life cycle, a secure infrastructure must be in place, from the sensor itself to the final database. This is essential, not only for regulatory compliance but also for building stakeholder confidence and safeguarding proprietary information.

### 3.5. Sensor-Based DPP Architecture

**Figure 2:**
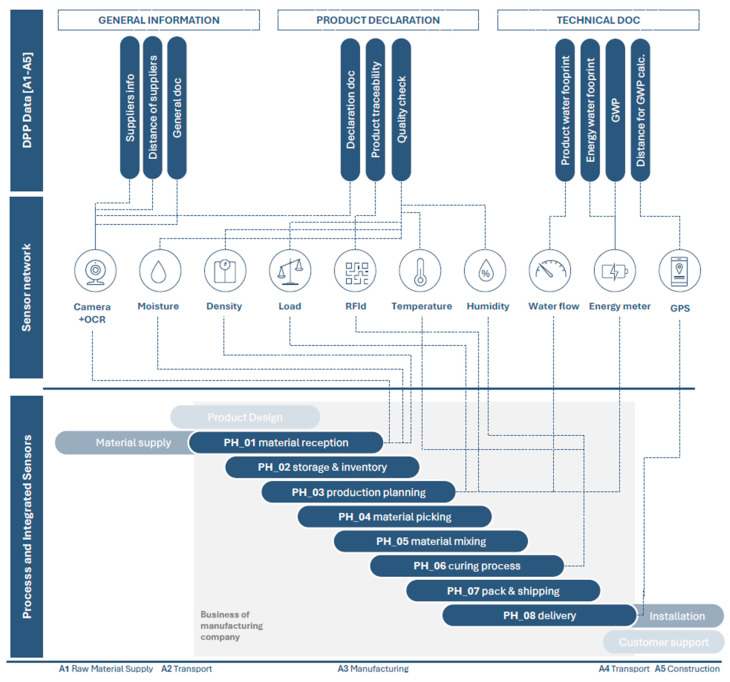
DPP’s fields filled through sensors and related data in different bio-block manufacturing phases.

**Figure 3:**
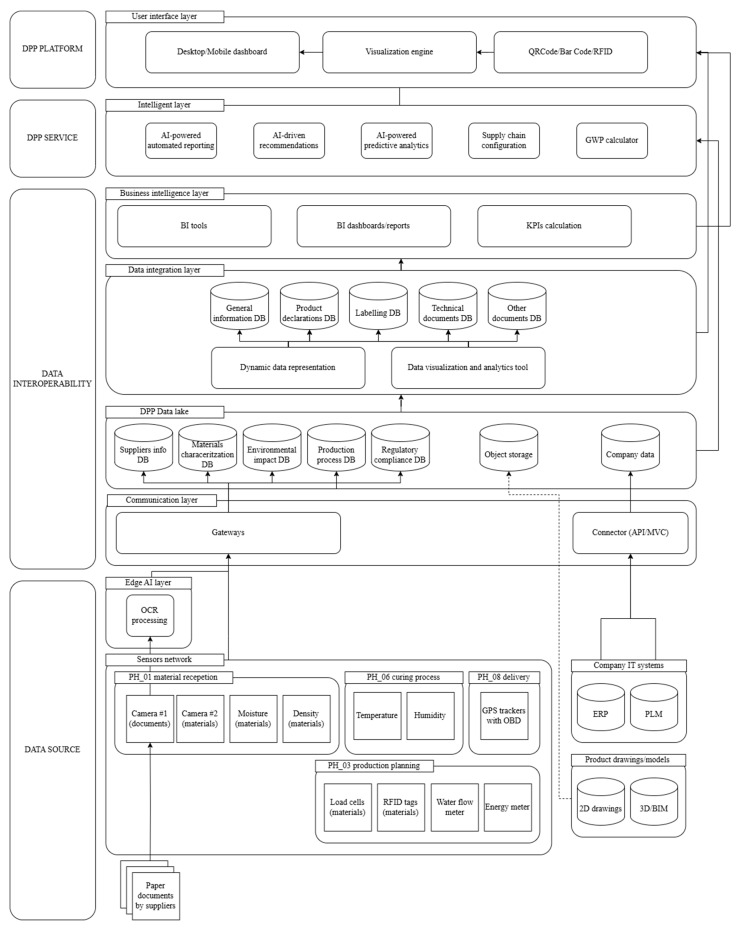
Sensor-based Digital Product Passport platform architecture.

Based on the analysis conducted, Figure 2 shows how specific sensors provide information to the DPP’s fields. The prevalent role that emerges is the usefulness of sensors to track environmental data, or to support the replacement of manual data entry and control of raw material in the supply chain. It utilizes a series of dedicated sensors that address the specific business needs identified in the preceding analysis. Figure 3 shows the sensor-based architecture for the DPP platform. The core of this architecture is the integration of sensors to track specific bio-block manufacturing phases and data from existing management software, such as PLM and ERP systems, with data collected from a new network using IoT and IIoT sensors. The priorities for implementation are environmental monitoring sensors for the critical 6-week curing process, along with resource consumption tracking for water and energy usage. This phased approach provides immediate value for DPP data requirements and regulatory compliance, while being mindful of the constraints of a low-technology manufacturing environment.

By organizing both existing and newly integrated data into a general and accessible data lake, the DPP can provide a comprehensive description of the product’s characteristics. This integrated data foundation enables the implementation of data analysis logic to generate clear KPIs related to the company’s and product’s impact. These insights are essential for initiating improvement plans and achieving a more granular and precise quantification of the product’s impacts, which align with decarbonization and circularity goals. The proposed system architecture focuses on minimum viable sensing solutions that can be integrated into existing production workflows without major infrastructure modifications. This approach maximizes early wins through established technologies while building organizational capacity for more complex integrations as digital maturity increases.

## 4. Discussion

Implementing a DPP is further complicated by the context of the bio-based industry. The production of bio-based building products using locally sourced, cross-sectorial residues requires traceability systems that can handle heterogeneous material inputs with variable quality characteristics. Current supplier documentation practices often lack standardized technical specifications, and materials are categorized into quality tiers through manual verification processes that compromise both efficiency and data reliability. This is particularly relevant for SMEs in the construction sector, where investment constraints and limited technical expertise necessitate carefully calibrated technology adoption strategies. The proposed approach is highly scalable and can be adapted to companies of different sizes. While this framework was designed for SMEs, its modularity allows it to be scaled up for larger enterprises by integrating more comprehensive data collection points and sophisticated analytical tools as needed. However, given the large number of SMEs and their low level of automation, future research should continue to focus on them, in order to make the supply chain in the construction sector completely transparent.

The results of the study indicate that a tiered approach to sensor deployment is the most effective strategy for these environments. Prioritizing environmental monitoring and process control sensors (Tier 1) for immediate deployment provides foundational data for DPP requirements and demonstrates a measurable return on investment (ROI) through improvements in energy efficiency and quality control. Subsequently, implementing document digitalization and ERP integration (Tier 2) can further streamline internal processes and data management. The final phase, advanced supply chain integration (Tier 3), which involves collaboration with external stakeholders like suppliers and logistics providers, represents the highest level of complexity, but also the greatest potential value for comprehensive traceability. This phased approach, which builds organizational capacity as digital maturity increases, provides a scalable framework for SMEs to adopt digital traceability systems without requiring substantial capital investment.

Based on the results presented in Table 1, a prioritization strategy emerges for sensor deployment in bio-block manufacturing DPP systems, revealing distinct implementation tiers based on complexity levels and value proposition for low-technology manufacturing environments:

- Tier 1 primary implementation (LOW complexity). User stories 12, 19, and 31 represent the foundation layer for sensor-integrated DPP systems, characterized by well-established technologies that can be deployed without external dependencies or major infrastructure modifications. The real-time recipe monitoring through load cells (ID-12) provides immediate value for dynamic recipe optimization and batch tracking, directly supporting the core manufacturing process. Modular sensor deployment (ID-19) enables gradual digitalization through plug-and-play standardized sensor packages for temperature, humidity, and flow monitoring, requiring minimal technical expertise while providing essential environmental data for the 6-week curing process. Direct environmental impact measurement (ID-31) through flow meters and energy meters delivers accurate LCA data that is crucial for competitive positioning and regulatory compliance, with straightforward implementation using ultrasonic sensors and current transformers.
- Tier 2 secondary implementation (MEDIUM complexity). User stories 03, 06, 11, and 27 constitute the enhancement layer, requiring more sophisticated integration but building upon established technologies. These implementations primarily face complexity from external variability factors rather than technological limitations. Document processing capabilities (ID-03, 11) through camera modules with edge AI for Optical Character Recognition (OCR) processing offer significant efficiency gains in data extraction, although success depends on the standardization of supplier document formats and templates. ERP integration (ID-06) through industrial IoT gateways provides substantial value for data standardization and workflow optimization, but complexity arises from the diversity of existing ERP systems and their varying integration capabilities. Automated material characterization (ID-27) delivers comprehensive quality control through multi-sensor stations, although effectiveness is constrained by the heterogeneous nature of agricultural waste materials and batch-to-batch variability.
- Tier 3 advanced implementation (HIGH complexity). User stories 01 and 15 represent the optimization layer, requiring external stakeholder coordination and presenting the highest implementation barriers, despite their significant potential value. Supplier integration (ID-01) through load cells and RFID tracking systems demands coordination with agricultural waste suppliers who may lack technical infrastructure or willingness to adopt sensor technologies. Transport tracking (ID-15) faces complexity from the variability of external logistics providers and the diversity of vehicle types and tracking systems used in regional supply chains.

In relation to the results from workflow analysis and sensors to be implemented for DPP, the analysis also reveals the chance to prioritize sensors for DPP integration based on implementation complexity and DPP value. The results, presented in tiers, are discussed below:

- Tier 1 primary implementation (LOW complexity). Phases PH_04, PH_06, and PH_07 constitute the core implementation foundation, offering immediate DPP value with manageable technical complexity. Material picking (PH_04) with automated data logging provides precise recipe adherence tracking, directly supporting batch-specific DPP records with straightforward technology deployment. Curing process monitoring (PH_06) represents the critical value driver for bio-block manufacturing, where distributed temperature and humidity monitoring during the 6-week ambient curing period generates essential environmental data for product quality certification and performance validation. Packaging and shipping (PH_07) through barcode systems and weight verification delivers immediate traceability value with minimal technical barriers, establishing the foundation for downstream supply chain tracking.
- Tier 2 secondary implementation (MEDIUM complexity). Phases PH_01, PH_02, and PH_08 represent the strategic enhancement tier, requiring moderate technical integration but delivering significant DPP data enrichment. Material reception (PH_01) through RFID readers and image recognition cameras enables automated supplier verification and material characterization, although complexity arises from integrating multiple recognition technologies with existing inventory systems. Storage and inventory (PH_02) provides real-time material tracking through RFID tags, offering operational efficiency gains while supporting accurate material life cycle documentation. Delivery tracking (PH_08) through GPS and RFID pallet verification extends DPP traceability to end customers, although implementation complexity increases with cloud-based delivery management platform integration.
- Tier 3 advanced implementation (HIGH complexity). Phases PH_03 and PH_05 constitute the optimization tier, requiring sophisticated technical integration and representing the highest complexity challenges. Production planning (PH_03) through API middleware linking PLM/ERP systems with real-time data feeds offers substantial competitive advantages through dynamic recipe optimization and supply chain efficiency, but demands extensive software development and multi-system integration capabilities that are considered risky, in particular for multiple IT systems connections. Production mixing (PH_05) through comprehensive process monitoring with industrial-grade environmental and energy consumption monitoring could suggest the adoption of SCADA for full integration (which is currently non-existent), although it provides critical process optimization data for competitive differentiation.

The tiered approach suggests prioritizing environmental monitoring and process control sensors (Tier 1) for immediate deployment, providing foundational data for DPP requirements, while demonstrating measurable ROI through energy efficiency and quality control improvements. Document digitalization and ERP integration (Tier 2) should follow, once internal processes are stabilized, focusing on standardizing supplier documentation formats to maximize OCR effectiveness. Supply chain integration (Tier 3) represents the final optimization phase, requiring collaborative agreements with suppliers and logistics providers to achieve comprehensive traceability. This implementation strategy acknowledges the reality of low-technology manufacturing environments where internal process control takes precedence over external supply chain coordination, while ensuring that each tier builds upon the previous layer’s data infrastructure and technical capabilities. The approach maximizes early wins through established technologies, while building organizational capacity for more complex integrations as digital maturity increases. Based on the tier classification, the sensor-based DPP architecture should prioritize specific sensors to support the collection of useful key data.

The conceptual architecture presented for a sensor-based DPP platform demonstrates a comprehensive framework for enabling traceability and compliance in the manufacture of low-technology bio-blocks. The specific scope of this research does not limit the scalability of the proposed approach to specific products or building systems. The case study was conducted only to clarify the specific features of the manufacturing sector for the construction industry. Its modular design integrates real-time sensor data, enterprise information systems, and semantic data modelling, enabling product passports to be updated dynamically throughout the entire life cycle, from raw material sourcing to delivery. The proposed architecture incorporates user stories and workflow requirements for DPP deployment in low-tech manufacturing sectors, such as the production of bio-based construction blocks. It integrates sensors with different levels of complexity at various stages of the product life cycle. Integrating sensing systems (e.g., moisture sensors, load cells, and energy meters) with legacy IT systems (e.g., ERP and PLM) provides a route to digitalization in contexts where resources are limited. This modular and scalable approach can also be adopted by SMEs. In particular, the architecture demonstrates the relevance of sensor networks in capturing key production data, representing a significant advancement from previous DPP systems that relied primarily on manually entered life cycle data, particularly for environmental impact analysis. This shift towards automated data acquisition specifically enables batch-specific DPP records, thereby improving the granularity and reliability of the data for life cycle inventory and carbon footprint assessment of the bio-block. Moreover, the architecture addresses the challenge of traceability of raw materials, which is particularly relevant for secondary raw materials sourced from agriculture. Including a camera for image recognition, moisture and density sensors, and RFID tagging allows precise documentation of the origin, properties, and compliance of materials at the reception stage, which is often missing in existing DPP frameworks that are mainly based on outdated supplier documentation.

The proposed multi-layered interoperability structure responds to the need for data harmonization across heterogeneous systems. This includes real-time sensor feeds, structured supplier documents, PLM data for product configurations, ERP data for administrative information, and regulatory compliance records.

The communication layer utilizes APIs, gateways, and connectors to enhance the system’s flexibility and scalability, enabling its adoption across various factory types without the need for a significant overhaul of infrastructure. While the architecture supports harmonization, flexibility, and scalability, it is technically feasible. However, it poses a risk in terms of the analysis of user stories for the interconnection of legacy IT systems (e.g., PLM and ERP), which could be a critical aspect of implementation and operation over time. Indeed, the involvement of multiple IT providers is considered a major risk for fully operative operations, and this must be a key aspect to consider when deploying the DPP platform. For this reason, autonomous sensor networks from legacy IT systems could be used to provide system redundancy, as this is considered less risky for end users. The results produced through the development of this theoretical framework will then be addressed and analyzed on a case-by-case basis in the next phases of the research. Aspects such as data format inconsistencies and data management systems’ interoperability will require specific in-depth analysis, particularly with legacy systems such as ERP, PLM, and MES, which represent a case-by-case scenario to be specifically investigated.

## 5. Conclusions

This research addressed the critical gap between emerging DPP regulatory requirements and the practical implementation constraints faced by low-technology manufacturing environments in the bio-based construction materials sector, in particular for bio-block manufacturers. Through a comprehensive case study of the bio-block manufacturing process, the sensor-integrated DPP architectures can be successfully implemented in traditionally under-digitized manufacturing environments, providing a scalable framework for enhancing traceability, circularity, and environmental accountability. The research demonstrates that sensor-integrated DPP systems represent a transformative opportunity for low-technology bio-based manufacturing, enabling simultaneous achievement of regulatory compliance, operational efficiency, and competitive differentiation. The three-tier implementation strategy provides a practical framework for gradual digitalization that respects the resource constraints and technical capabilities of SMEs while building toward comprehensive supply chain transparency. The sensor-integrated DPP architecture presented in this research thus represents not merely a technological solution, but a strategic framework for enabling the digital and green transition in traditionally under-digitized manufacturing sectors, demonstrating that sustainability goals and business competitiveness can be simultaneously achieved through thoughtful technology integration. Implementing a sensor-based DPP architecture that systematically organizes and shares key product life cycle information with key stakeholders throughout a product’s lifespan is crucial. This enables the accurate assessment of materials’ environmental and performance impacts, making it an essential practice for driving company improvement strategies that are aligned with the decarbonization and circularity goals.

Despite the low level of digitalization prevalent among EU manufacturing SMEs in the construction sector, the case study demonstrates that significant advancements in integrated data management are achievable. These can be accomplished through the strategic organization of existing data from current management software and the implementation of low-cost sensing technologies. From this perspective, the cost for the implementation of a sensor technology should be viewed as a strategic capital investment for companies, with its returns and benefits evaluated over a medium- to long-term horizon. Such data is essential for ensuring the greater granularity and precision needed for comprehensive visibility of a product’s characteristics, its supply chain, and the objective quantification of its impacts. Future research should focus on economic analysis of implementing scalable digital architectures tailored to specific business needs. The researchers acknowledge that, for companies already utilizing advanced systems such as SCADA, implementing additional sensors specifically for DPP requirements may represent more of an academic exercise than a practical business necessity. On the other hand, for low-technology manufacturing environments where sensor integration simultaneously addresses production process optimization and regulatory compliance requirements it can represent an add-on in company insights and in tracking key environmental information for DPP.

Furthermore, the proposed architecture’s automation of data collection and digitalization offers substantial benefits. It reduces the administrative burden and potential errors associated with manual technical documentation, while also providing new analytical tools for operational planning, business optimization, and sustainable reporting.

The systematic analysis of the bio-block manufacturing workflow identified strategic implementation priorities across three complexity tiers, emphasizing the role of sensors for environmental monitoring with the highest value and lowest complexity entry point for sensor deployment in a low-tech factory such as the bio-block facility. This finding directly addresses the unique characteristics of bio-based materials manufacturing, where natural processes require continuous monitoring to ensure product quality and performance validation. While the system is designed for bio-block, the core architecture is adaptable to other product categories, particularly those involving batch production, secondary material use, or distributed supply chains (e.g., ceramics, composites, agri-based polymers). However, the utilization of this DPP framework integrated with sensors for other bio-based products requires its adaptation to specific production characteristics. While the DPP platform architecture remains replicable across different bio-based materials, sensor setup must be defined based on the specific production processes of each bio-based material. Different bio-based products, varying in the raw materials used and the production processes, necessitate sensor configurations designed for specific cases, although these can be integrated with the information and data that can be collected within the DPP system. This finding has significant implications for the broader bio-based materials sector. The modular sensor architecture developed in this research provides a foundational framework and methodology that can be adapted for various bio-based production processes, while maintaining DPP data standardization and interoperability.

## Figures and Tables

**Table 1:** Sensors identified and DPP features enabled from user stories analysis.

Id	DPP Feature	Sensor Implementation	Implementation Complexity
US_01	Editable input for supplier data, database field for supplier-specific info	Load cells for automated material weight tracking from suppliers RFID tags for traceability chain for origin verification	HIGH—Involvement of material suppliers installing and adopting sensor
US_03	Document upload with auto data extraction	Camera modules with edge AI for OCR processing for technical data extraction from supplier documents	MEDIUM—Well-established technology, but high variability of documents processed with multiple templates and formats dependent on suppliers
US_06	Automated data synchronization	Gateway devices for industrial IoT gateways for sensor data aggregation and ERP API integration	MEDIUM—Multiple ERP integration with closed system and data standardization
US_11	Document management	Camera modules with edge AI for OCR processing, digitalization of physical documents and organization	MEDIUM—Well-established technology, but high variability of documents processed with multiple templates and formats dependent on suppliers
US__12	Real-time recipe monitoring	Load cells for component weighing measurement before mixing	LOW—Well-established technology to enable dynamic recipe optimization and batch tracking before production stage
US_15	Automated transport tracking	GPS trackers with OBD for vehicle tracking modules for delivery routes, fuel consumption monitoring for accurate transport emission	HIGH—Transport is an external service with variability in service providers and vehicles used
US_19	Modular sensor deployment	Plug-and-play sensor modules with standardized sensor packages (temperature, humidity, flow) with wireless connectivity for different process workflows	LOW—Well-established technology to enable modular and gradual digitalization without major infrastructure changes
US_27	Automated material characterization	Multi-sensor characterization station with combined camera + moisture + density sensors for comprehensive material analysis	MEDIUM—Well-established technology for automating quality control and ensuring consistent material standards, but with limitations in tracking multiple non-homogeneous batches
US_31	Direct environmental impact measurement	Flow meters and energy meters with ultrasonic flow sensors for water usage, current transformers for energy consumption monitoring	LOW—Well-established tech with accurate environmental data for competitive LCA reporting

**Table 2:** Bio-block manufacturing workflow with data collection framework.

Id	Phase	Technical Feature	Sensor Implementation	Implementation Complexity
PH_01	Material reception	Unload and inspect incoming raw materials, verify against supplier documentation, create internal handling unit, characterize material granularity with sample check	Material and quality recognition. RFID/QR code readers for supplier docs and internal units. Image recognition cameras for visual inspection; laser diffraction for particle size analysis	MEDIUM—Requires integration of readers and cameras with inventory systems. Advanced sensors for granularity are more complex
PH_02	Storage and inventory	Store raw materials in warehouse, update inventory records and collect paper-based documentation	Product inventory. RFID tags on handling units; ultrasonic/infrared sensors for bin-level monitoring; smart scales for weight tracking	MEDIUM—Initial setup of tags and readers, but provides real-time inventory. Bin-level sensors are a simple, low-cost option
PH_03	Production planning	Create production schedules and orders based on customer requirements and material availability, optimize bio-block formulation recipes	Integration of API/middleware for linking PLM/ERP with sensor data; AI-based optimization software for material utilization	HIGH—This requires sophisticated software and integration with multiple systems, including real-time sensor data feeds
PH_04	Material picking	Pick materials from storage based on production orders, weigh materials according to recipe specifications	Smart scales with automated data logging; barcode/QR code scanners for verification; pick-by-light or augmented reality for guidance	LOW/MEDIUM—Smart scales are straightforward. Implementing pick-by-light or AR is more complex but can greatly improve accuracy
PH_05	Production mixing	Assemble materials using large mixers, forming through stamping plant, mold filling via hoppers, pre-stacking and transport to pre-curing chambers	Process monitoring with temperature, humidity, for quality control; load cells for accurate material mixing; motor sensors for energy consumption	MEDIUM/HIGH—Requires robust, industrial-grade sensors in a harsh environment. Integration with a centralized control system (SCADA) would be necessary for full benefit
PH_06	Curing process	Natural ambient temperature curing with protection from external conditions, monitoring environmental parameters during 6-week curing period	Distributed temperature and humidity sensors in curing chambers for product quality control	MEDIUM—Off-the-shelf wireless sensors for temperature/humidity are widely available and easy to deploy
PH_07	Packaging and shipping	Pack finished bio-blocks, create shipping labels and documentation, prepare for transport	Tracking product with barcode or QR code printers/scanners for final product tracking	LOW—Barcode systems are standard. Integrating weight sensors is a minor addition to an existing line
PH_08	Delivery	Load products onto trucks or other means of transportation for delivery to end customers or construction sites	Tracking delivery trucks by GPS; RFID on final pallets for automated loading/unloading verification	MEDIUM—GPS tracking is a mature technology. Integrating it with an internal system and using RFID for automated checks adds complexity

**Table 3:** Data inventory from workflow phase.

Id Phase	Dataset Name	Data	Type	Data Origin	Data Storage Methodology
PH_01	Supplier information	Supplier identity, location, transport distance, material availability, declaration of conformity, technical sheets, EPD documentation	S/SS	Supplier information	M: Manual filing and spreadsheet records F: Text documents, spreadsheets F.S.: Per delivery batch F.R.: As needed for production planning F.U.: When supplier information changes
PH_02	Material characterization	Hemp fiber specifications, olive stone characteristics, almond shell properties, lime composition, granulometry data, dust residue levels, density measurements	S	Supplier technical sheets, manual laboratory testing	M: Spreadsheet records, physical sample storage F: Spreadsheets, text documents F.S.: Per batch F.R.: Daily for production planning F.U.: Per batch received
PH_05	Environmental impact data	GWP values, LCA data, carbon sequestration potential, water usage coefficients, transport emissions	S/SS	LCA databases, supplier EPD, internal calculations	M: Manual entry in spreadsheets F: Spreadsheets, text documents F.S.: Annual/semi-annual updates F.R.: Monthly for reporting F.U.: When methodology updates
PH_05	Production process data	Temperature monitoring, humidity levels, water usage, energy consumption, mixing time, forming parameters, curing chamber conditions	D	Not collected	M: Not collected F: Not collected F.S.: Not collected F.R.: Not collected F.U.: Not collected
PH_05	Quality control	Batch testing results, compliance verification, non-conformity reports, corrective actions	D	Laboratory testing, visual inspection	M: Manual reports, spreadsheet tracking F: Text documents, spreadsheets F.S.: Per batch F.R.: Weekly quality reviews F.U.: Per batch
PH_05	Regulatory compliance	CSRD reporting data, environmental permits, safety certifications, audit records	S/SS	Regulatory authorities, internal compliance	M: Document filing, spreadsheet tracking F: Text documents, spreadsheets F.S.: Annual/as required F.R.: Quarterly reviews F.U.: Annual/as required

Type: S = static; SS = semi-static; D = dynamic. Data storage methodology: M = storage methodology; F = data format; F.S. = frequency sampling; F.R. = frequency reading; F.U.: frequency data updating.

**Table 4:** Manual to automated data collection workflow.

Id Phase	Data Collection Point	Current Method	Proposed IoT Solution	Sensor Type	Data Frequency
PH_01, PH_05	Material weight tracking	Manual scale readings	Automated weight monitoring	Load cells, digital scales	Per material handling event
PH_05	Water usage in mixing	Not collected	Automated water consumption tracking	Flow sensors	Per mixing cycle
PH_05	Energy consumption	Monthly utility bill analysis	Real-time energy monitoring	Current transformers, smart meters	Continuous (1′ intervals)
PH_05	Production line status	Manual production logs	Automated process monitoring	Proximity sensors, current monitoring	Real-time
PH_06	Curing chamber temperature	Not collected	Automated temperature monitoring	Temperature sensors	Continuous (15′ intervals)
PH_06	Curing chamber humidity	Not collected	Automated humidity monitoring	Humidity sensors	Continuous (15′ intervals)
PH_07	Environmental storage conditions	Not collected	Continuous environmental monitoring	Combined temp/humidity sensors	Continuous (15′ intervals)
PH_08	Material transport tracking and documentation	Manual documentation	RFID/barcode tracking	RFID readers, barcode scanners	Per movement event

## Data Availability

No new data were created or analyzed in this study. Data sharing is not applicable to this article.

## Abbreviations

The following abbreviations are used in this manuscript:

CRUD	Create, Read, Update and Delete
DB	Database
DPP	Digital Product Passport
EDP	Multidisciplinary Digital Publishing Institute
ERP	Enterprise Resource Planning
GPS	Global Positioning System
GWP	Global Warming Potential
IoT	Internet of Things
IIoT	Industrial Internet of Things
OBD	On-Board Diagnostic
OCR	Optical Character Recognition
PLM	Product Life Cycle Management
ROI	Return on Investment
SCADA	Supervisory Control and Data Acquisition
UI	User Interface
UX	User Experience

## Appendix A

The table below reports the extended results of interviews with user stories and the related user requirements, technical requirements, and KPIs. Legend:

- Company Direction—CD;
- Chief Financial Officer—CFO;
- Production Engineer—PE;
- Project Manager—PM.

**Table A1:** User stories from interview adopted for DPP platform design.

Id	As a… (Type of User)	I Want to… (Perform a Task)	So That I Can… (Achieve This Goal)	User Requirement	Technical Requirement	KPI (Key Performance Indicator)	Means of Verification
01	CD	Reduce use of hemp	Optimize scores for use of local supply chain despite lower quantities	Data-driven material sourcing optimization	Material scoring algorithm linked to local sourcing	% increase in local scoring under set thresholds	DPP-generated reports; scoring analytics
02	CD	Use a platform suitable for non-specialists	Enable multiple staff to contribute to GWP/DPP creation	Intuitive UI; guided data entry	Role-based access; simplification of form fields	# of users actively compiling DPPs	User logs; platform analytics
03	CD	Upload technical data sheets from suppliers	Avoid repetitive manual data entry	Upload function + auto-fill fields	File parser to extract info from PDFs or Excel	Time saved per DPP compilation	Timing tests; user interviews
04	CD	Automatically classify and search products	Manage large product lists more efficiently	Smart search and classification	NLP-based search from tech sheets + DPP metadata	Search speed and accuracy	UX testing; search success rate
05	CD	Know what specific data is required	Reduce time lost learning how to fill forms	Clear form instructions and mandatory field indicators	Form validation + info pop-ups	Time to complete DPP form	Timing analysis; feedback survey
06	CD	Connect DPP with existing ERP systems	Reduce IT overhead and ensure cost–benefit ratio	API integration only for companies with hundreds of DPPs	Scalable API connector	# of successful ERP–DPP integrations	API usage logs; integration testing
07	CD	Use Excel-based forms	Input data more comfortably with all data in one view	Option to export/import from Excel	Excel template + import engine	% of forms submitted via Excel	Submission format statistics
08	CD	View and modify all data later	Ensure flexibility in data management	Save-and-edit function	CRUD access to data; autosave	% of revisited DPPs	User logs; modification timestamps
09	CD	Break down only missing info for completion	Reduce cognitive load and increase completion rate	Progressive disclosure	Conditional logic in form engine	Form completion rate	Form analytics
10	CD	See how long a questionnaire takes	Avoid uncertainty and fatigue in data entry	Estimated time to complete before starting	Timer display based on complexity score	User satisfaction score	Survey; platform feedback
11	CD	Classify and locate supplier documents quickly	Reduce time spent searching for documents	Document categorization system	Tagging and document indexing system	Time saved in document retrieval	User testing; document access logs
12	CD	Modify product recipe and update DPP accordingly	Keep the DPP dynamic and customizable for innovation	Open platform with editable product inputs	Editable DPP fields and version tracking	# of modified DPPs	Audit logs; change tracking
13	CD	Exclude specific sensitive info from being shown to clients or competitors	Protect business-critical data	Lock/unlock visibility toggle per data field	Role-based data visibility; data masking	Possibility of locked data fields	Visibility settings log
14	CD	Show raw materials but hide detailed logistic/quantity info, unless unlocked deliberately	Control supply chain transparency selectively	Toggle to unlock supply chain info when needed	Conditional data visibility; user-defined permissions	Access log to detailed info	Admin-level user logs
15	CD	Show transport data in a simplified form like annual averages	Avoid excessive complexity while still being transparent	Summary metrics or default averages	Backend logic for annual transport footprint	Accuracy of CO_2_ transport data	Calculated value vs external LCA tool
16	CD	Avoid forced integration with ERP unless truly necessary	Reduce IT complexity for SMEs	Optional ERP integration only above a certain threshold	Tiered integration framework	Integration rate by company size	Tech adoption statistics
17	CD	Distribute different data (e.g., DoP, tech sheet) to different stakeholders	Manage communication efficiently across the value chain	Role-specific document export	Custom PDF/CSV generation for different actors	Stakeholder satisfaction	Export logs; interview feedback
18	CFO	Ensure privacy compliance	Avoid legal issues with sensitive transport documentation (DDT)	GDPR-compliant data handling	Secure data protocols; encryption; role-based access	Zero data breach incidents	GDPR audit; penetration testing
19	CFO	Stay compliant with evolving regulations	Ensure long-term use of the tool and avoid regulatory penalties	Compliance tracking module	Regulatory update tracker + compliance database	Number of updates pushed	Update logs; user notification dashboard
20	CFO	Start manual input, then switch to automation when needed	Scale tool adoption based on needs	Two-phase deployment (manual first, then API)	MVP first; scalable integration module	Transition success rate	System usage and cost-saving assessment
21	CFO	Obtain assistant suggestions for data entry (e.g., CO_2_ values)	Simplify form completion	Intelligent assistant with prefilled field suggestions	CO_2_ and water database with look-up support	Accuracy of suggestion engine	Form validation logs; error rate
22	CFO	Save drafts and return later with virtual assistant support	Improve usability and reduce friction	Draft mode and progress-saving	Local storage/cache; cloud-based autosave	% of saved/incomplete DPPs	Draft analytics
23	CFO	Use Excel to manage and copy/paste product data	Streamline product-level data compilation	Excel template designed for mass data entry	Multi-line import and product cloning	# of product rows per upload	Upload logs
24	CFO	View all required info at first glance	Prepare necessary data beforehand	Overview dashboard or checklist	Summary card or table with required fields	Time to first data input	First-touch user experience survey
25	CFO	Understand what the assistant will ask next	Be mentally prepared for data input	Assistant roadmap preview	Wizard-like interface with visible steps	Completion drop-off rate	Wizard usage analytics
26	CFO	Allow any employee to upload documents and auto-fill forms	Reduce training needs and distribute task load	Document upload with auto-parsing	AI extraction from common formats (PDF, Excel)	Error rate of extracted data	QA reports; training/testing documents
27	PE	Use Excel for any operation possible	Work with a familiar tool and reduce time spent on new platforms	UI/UX similar to Excel	Design of interactions and functionalities of “data entry” similar to Excel	User satisfaction	Feedback survey; adoption rate
28	PE	Insert material characterization per supplier in system	Provide reliable data and traceability	Editable input for supplier data	Database field in DPP for supplier-specific info	% of products with complete supplier profiles	System audit; database query
29	PM	Build a supplier database to avoid ERP integration	Maintain agility and reduce complexity	Modular database per supplier	Decentralized data storage with tagging	Number of supplier DBs created	DB log; admin dashboard
30	PM	Add a dedicated module for technical data, separate from PLM	Customize tech data without overloading main system	Lightweight PLM-alternative	Custom module for material specs	Usage rate of tech module	Module logs
31	PM	Include water usage and carbon sequestration data	Capture differentiating LCA factors	Expand LCA scope	Extra fields for water use and sequestration	LCA completeness score	LCA form completeness analytics
